# GM-CSF Increases Mucosal and Systemic Immunogenicity of an H1N1 Influenza DNA Vaccine Administered into the Epidermis of Non-Human Primates

**DOI:** 10.1371/journal.pone.0011021

**Published:** 2010-06-08

**Authors:** Peter T. Loudon, Eric J. Yager, Debbie T. Lynch, Amithi Narendran, Cristy Stagnar, Anthony M. Franchini, James T. Fuller, Phil A. White, Julia Nyuandi, Clayton A. Wiley, Michael Murphey-Corb, Deborah H. Fuller

**Affiliations:** 1 PowderMed Ltd., Oxford, United Kingdom; 2 Center for Immunology and Microbial Disease, Albany Medical College, Albany, New York, United States of America; 3 Recombiworks, Ltd., Clifton Park, New York, United States of America; 4 Department of Medical Microbiology and Molecular Genetics, University of Pittsburgh School of Medicine, Pittsburgh, Pennsylvania, United States of America; 5 Division of Neuropathology, Department of Pathology, University of Pittsburgh Medical Center, Pittsburgh, Pennsylvania, United States of America; Karolinska Institutet, Sweden

## Abstract

**Background:**

The recent H5N1 avian and H1N1 swine-origin influenza virus outbreaks reaffirm that the threat of a world-wide influenza pandemic is both real and ever-present. Vaccination is still considered the best strategy for protection against influenza virus infection but a significant challenge is to identify new vaccine approaches that offer accelerated production, broader protection against drifted and shifted strains, and the capacity to elicit anti-viral immune responses in the respiratory tract at the site of viral entry. As a safe alternative to live attenuated vaccines, the mucosal and systemic immunogenicity of an H1N1 influenza (A/New Caledonia/20/99) HA DNA vaccine administered by particle-mediated epidermal delivery (PMED or gene gun) was analyzed in rhesus macaques.

**Methodology/Principal Findings:**

Macaques were immunized at weeks 0, 8, and 16 using a disposable single-shot particle-mediated delivery device designed for clinical use that delivers plasmid DNA directly into cells of the epidermis. Significant levels of hemagglutination inhibiting (HI) antibodies and cytokine-secreting HA-specific T cells were observed in the periphery of macaques following 1–3 doses of the PMED HA DNA vaccine. In addition, HA DNA vaccination induced detectable levels of HA-specific mucosal antibodies and T cells in the lung and gut-associated lymphoid tissues of vaccinated macaques. Importantly, co-delivery of a DNA encoding the rhesus macaque GM-CSF gene was found to significantly enhance both the systemic and mucosal immunogenicity of the HA DNA vaccine.

**Conclusions/Significance:**

These results provide strong support for the development of a particle-mediated epidermal DNA vaccine for protection against respiratory pathogens such as influenza and demonstrate, for the first time, the ability of skin-delivered GM-CSF to serve as an effective mucosal adjuvant for vaccine induction of immune responses in the gut and respiratory tract.

## Introduction

Current influenza vaccines protect against homologous viruses but are less effective against antigenic variants and provide little, if any, protection against a different subtype. In the event of a pandemic, existing vaccines may be ineffective because the manufacturing process requires at least 6 months from identification of the pandemic strain to distribution which is insufficient time to prevent wide-scale morbidity or mortality. New vaccine strategies are therefore needed that can both accelerate production and provide broader spectrum protection.

DNA vaccines offer a theoretical advantage over conventional vaccine strategies in that they can be more rapidly developed in the event of an influenza pandemic following the identification of the newly emerging strain. Once the sequence of the relevant hemagglutinin gene is known, a DNA vaccine can be synthesized to express the antigen within a week and rapidly scaled up using bacterial cell culture. In contrast to conventional approaches there is no need for live influenza virus or large quantities of eggs, and DNA vaccines could be deployed earlier in the pandemic to more effectively reduce morbidity and mortality. It is also straightforward to develop designer vaccines that contain consensus sequences capable of inducing antibody that cross-neutralizes drifted or shifted strains of influenza [1]. Importantly, DNA vaccines have afforded significant protection against heterologous challenges with genetically drifted strains (e.g., heterosubtypic immunity) in animal models, an outcome that may be due to their ability to also induce robust CD8+ cytotoxic T lymphocyte (CTL) responses against more conserved regions of the virus [1], [2], [3], [4], [5]. Such responses may not prevent infection *per se*, but could reduce viral load, mediate faster viral clearance, reduce morbidity, and, importantly for a viral pandemic, prevent mortality.

Particle mediated epidermal delivery (PMED) of DNA vaccines involves the use of a needle-free device to deliver plasmid vaccines directly into cells of the epidermis of the skin including both non-professional (i.e. keratinocytes) and professional antigen presenting cells (i.e. Langerhans cells) [6], [7], [8]. PMED influenza DNA vaccines have been extensively studied in relevant animal models including mice, ferrets, and swine and have been shown to induce protective antibody and cytotoxic T cell responses that provided complete protection against both homologous and drifted strains of influenza A viruses [9], [10], [11], [12], [13], [14], [15], [16]. Importantly, PMED-based DNA vaccination may prove to be particularly advantageous over other DNA vaccine delivery methods due to its apparent ability to induce mucosal immune responses. Strong mucosal immune responses induced in both the gut and lung by PMED DNA vaccines have contributed significantly to enhanced and complete protection against mucosal challenges with both AIDS viruses and influenza [13], [14], [17], [18], [19]. The ability of PMED DNA vaccination to elicit mucosal immunity is explained by the finding that the epidermis of the skin can serve as a potent inductive site for mucosal responses in distal mucosal sites such as lung and gut [20], [21], [22], [23].

PMED influenza DNA vaccines have shown considerable promise in the clinic. In contrast to DNA vaccines employing intramuscular needle injection where milligram doses of plasmids induced poor responses in humans [24], [25], [26], [27], PMED delivery of 1–4 µg doses of an influenza DNA vaccine stimulated protective levels of antibody in 100% of the vaccinated subjects, and responses exceeded the minimum criteria for licensure established by the Committee for Human Medicinal Products [28]. In a subsequent phase 1 clinical study where subjects were vaccinated with a trivalent PMED DNA influenza vaccine and then challenged with a controlled dose of an H3 influenza virus, the vaccine was well tolerated, induced anti-hemagglutinin (HA) antibody responses and provided protection against challenge with a vaccine efficacy of 53% against upper respiratory tract infection [29].

Despite these promising data there is still a need to increase the potency of PMED influenza DNA vaccines in the clinic to provide protection at or above the 70% protection level seen with trivalent inactivated influenza vaccines [30]. One method to enhance potency is to include a genetic adjuvant in the formulation of the DNA vaccine. Several reports have shown that co-administration of an adjuvant plasmid expressing the cytokine GM-CSF (granulocyte macrophage-colony stimulating factor) substantially increased both the immunogenicity and protective efficacy of DNA vaccines in mice and nonhuman primates [31], [32], [33], [34], [35], [36]. GM-CSF is a pleiotropic cytokine that stimulates the development, proliferation, and maturation of dendritic cells and monocytes. These antigen presenting cells are critical mediators for vaccine induction of immune responses. GM-CSF has been shown to enhance the ability of influenza DNA vaccines to induce antibody responses and protection in mice [37], [38], but not in the context of influenza DNA vaccines in either the clinic or a relevant large animal preclinical model. In addition, the capacity for GM-CSF to enhance the ability of DNA vaccines to induce mucosal responses has not been explored. We reasoned that GM-CSF may be a particularly potent adjuvant for augmenting the immunogenicity of PMED in both larger animals and the clinic as PMED can deliver both the vaccine plasmid and adjuvant plasmid directly into the same antigen presenting cells of the skin [6], [39]. In this study we provide evidence that co-delivery of GM-CSF with an H1N1 influenza DNA vaccine using a disposable PMED clinical device substantially increased DNA vaccine potency for induction of both neutralizing antibody and T cell responses in the highly relevant preclinical nonhuman primate model. Furthermore, we show for the first time that GM-CSF, when administered to the epidermal layer of the skin, can serve as a highly effective adjuvant for enhancing mucosal T cell responses in both the lung and gut of rhesus macaques.

## Results

### Particle-mediated epidermal delivery of influenza DNA vaccines into nonhuman primates using the ND10 clinical delivery device

**Figure 1:**
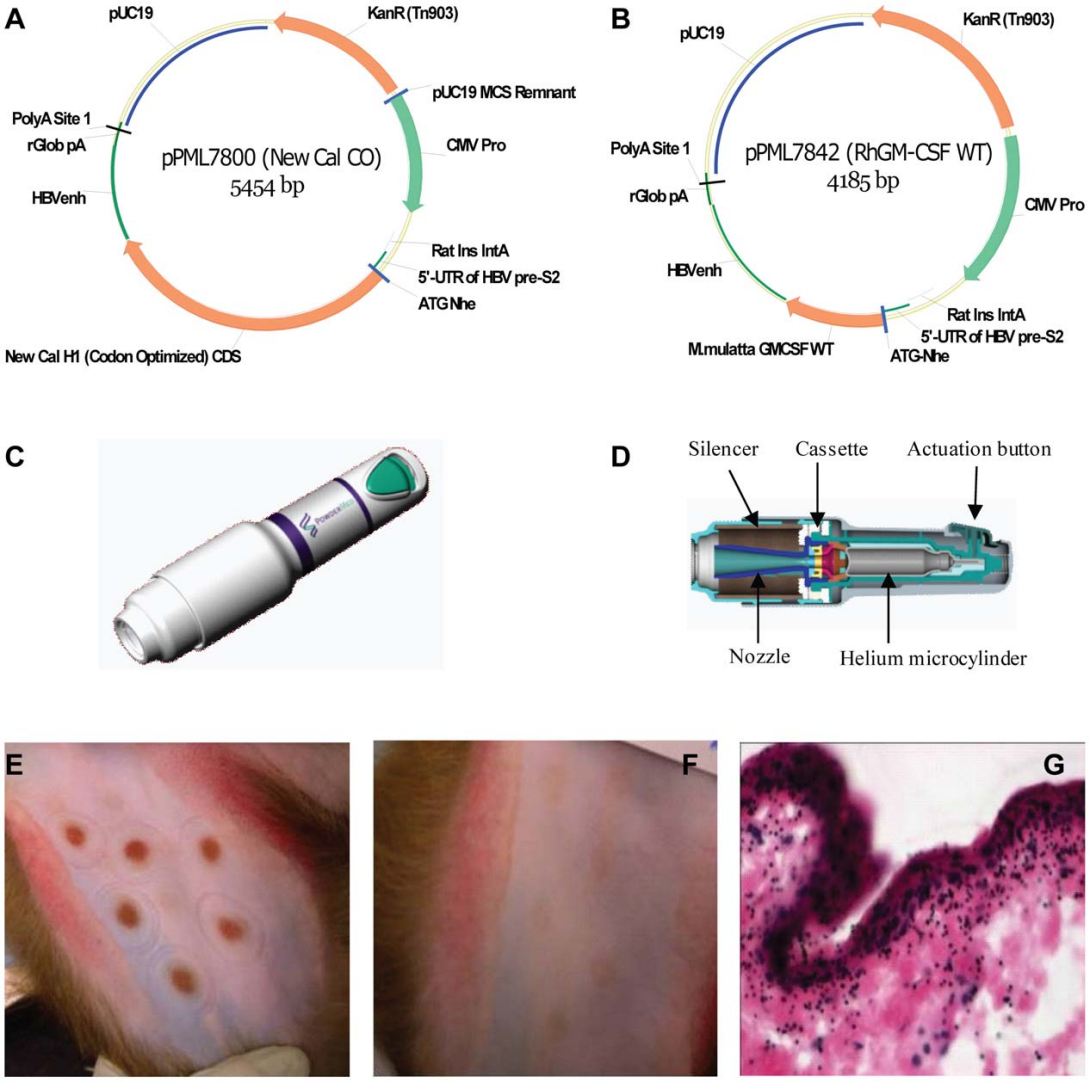
Particle-mediated DNA vaccine delivery into the epidermis of rhesus macaques using the disposable commercial prototype ND10 device. Plasmid DNA encoding (**A**) the gene for influenza A/New Caledonia/20/99 haemagglutinin (HA) was administered alone or in combination with (**B**) a plasmid encoding the gene for rhesus macaque granulocyte-macrophage colony stimulating factor (rhGM-CSF) co-formulated onto 1–3 µM gold particles. (**C**, **D**) The ND10 delivery device consists of a cassette containing 2.0 µg plasmid (1.8µg HA DNA+0.2µg GM-CSF DNA) coated onto 1.0 mg of gold particles, a safety catch that is released when the device is held firmly against the skin surface, and an actuation button that breaks the tip off a gas microcylinder and releases helium at high pressure. Release of the helium ruptures the cassette membrane, entrains the DNA-coated gold particles into the helium jet, and propels them directly into cells in the skin. (**E**) Vaccinations were targeted to the skin located on the upper inner thigh adjacent to the inguinal lymph node. Immediately following vaccination, vaccination sites are easily visualized as red (erythema) targets in the skin. (**F**) The erythema is transient (24 hours) and vaccination sites faded but were still discernible at 8 weeks post-vaccination. (**G**) Shown is gold particle penetration into the epidermal and dermal skin layers in a representative histological cross-section of a skin biopsy collected 10 minutes after ND10 delivery.

DNA plasmids encoding the H1N1/New Caledonia hemagglutinin (HA) gene alone (2.0 µg) (Figure 1A) or co-formulated with rhesus macaque GM-CSF DNA (1.8 µg HA DNA vaccine +0.2 µg GM-CSF) (Figure 1B) were precipitated onto 1.0 mg of 1–3 µM gold particles. The commercial prototype ND10 device is a disposable, single use delivery device (Figure 1C) that contains a helium gas microcylinder, an expansion chamber with a cassette containing the DNA vaccine-coated gold particles, nozzle, and actuation button (Figure 1D). To administer the vaccine, the nozzle of the device cover is pushed down against the skin. This action over-rides the button safety catch allowing the button to be depressed only when the nozzle is in position. Pressing the button breaks the tip off the gas microcylinder, releases the helium gas, bursts the vaccine cassette, and forces the particles down the nozzle and into the epidermal layers of the skin (Figure 1D). Delivery into the skin of the inner thigh of rhesus macaques resulted in transient erythema at the site of inoculation (Figure 1E). The redness resolved within 24 hours after delivery and then faded by 8 weeks post-administration (Figure 1F). This transient skin reaction to PMED delivery using the ND10 device in nonhuman primates is consistent with skin reactions to PMED reported in humans [40]. Histological analysis of the skin site in biopsies excised 24 hours post-delivery showed significant particle penetration in the epidermis of the skin (Figure 1G), a result that is consistent with the level of penetration required for optimum immunogenicity of PMED DNA vaccines [6], [7].

Rhesus macaques received particle-mediated epidermal immunizations over the inguinal lymph node with the ND10 device. Each macaque received a total of 12.0 µg/dose of a DNA vaccine expressing the influenza A hemagglutinin gene (HA) either alone (N = 6) or in combination with a second plasmid expressing rhesus macaque GM-CSF (HA+GM-CSF, N = 6). The 12.0 µg DNA vaccine dose was accomplished by delivering 6 targets per dose consisting of 1.0 mg gold and 2.0 µg of DNA per target. Each macaque received 3 doses (prime and 2 boosts) administered 8 weeks apart (weeks 0, 8, 16). The dose, immunization regimen and 9∶1 ratio of DNA vaccine to genetic adjuvant used in these studies were previously determined to be optimum in rhesus macaques and mice [41], [42], [43]. Blood, nasal, and tracheal secretions were collected after each dose to analyze serum and mucosal antibody and systemic T cell responses. Bronchioalveolar lavages were collected after the 2^nd^ dose to analyze mucosal antibody and T cell responses. Lung tissue and jejunum were also collected 8 weeks after the final dose to analyze mucosal T cell responses.

### GM-CSF increases serum antibody responses

**Figure 2:**
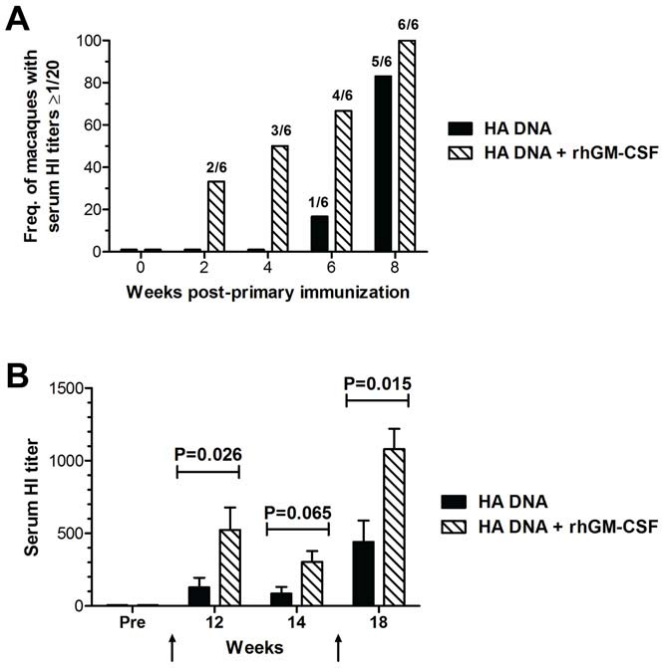
Induction of virus neutralizing antibodies in the sera of macaques following immunization with either the unadjuvanted, or rhGM-CSF adjuvanted, PMED HA DNA vaccine. Influenza A/New Caledonia/20/99 virus-neutralizing antibodies present in serum of macaques at the indicated times following vaccination with HA DNA alone (black bars) or HA DNA co-delivered with rhGM-CSF (hatched bars) were measured using a standard hemagglutination inhibition (HI) assay. (**A**) Serum HI titers measured in individual animals at the indicated times following the primary immunization at week 0. Bars indicate the frequency of animals in each group (n = 6) exhibiting a HI titer greater than or equal to 1/20. Numbers above each bar indicate the actual number of macaques in each group used to calculate the frequencies. (**B**) Serum HI titers were measured in vaccinated animals following boosting doses of vaccine at weeks 8 and 16 (indicated by the arrows). Bars indicate the means (± SEM) calculated for each group of at the indicated times following vaccination. Indicated P values were determined by the Mann-Whitney U test (two-tailed).

Current influenza vaccines protect via induction of neutralizing antibody specific for the viral HA. Serum from macaques as various times post-vaccination was analyzed for the presence of neutralizing antibodies by the hemagglutinin inhibition assay (HI). Analysis of serum HI titers after the first immunization showed that co-administration of GM-CSF accelerated the development of A/New Caledonia/20/99 virus-neutralizing antibodies. By 4 weeks after a single immunization, 50% (3/6) of the animals in the GM-CSF group had detectable serum HAI titers (≥1/20) whereas antibody was not yet detectable in any of the 6 animals in the unadjuvanted DNA vaccine group (Figure 2A). By 8 weeks and prior to the first boost, 5 of 6 animals in the HA DNA only group (25 mean titer, ±5.0 SEM) and all 6 animals in the HA DNA+GM-CSF group (40 mean titer, ±8.9 SEM) had detectable HI titers. Serum HI titers in both groups were increased following the first boost to levels that clearly exceeded the minimum titer associated with protection in humans (titer >40 HI). However, the mean HI titer in the GM-CSF adjuvanted group after the first boost was significantly higher than in HA DNA only group (Figure 2B). The GM-CSF adjuvanted group also maintained a significantly higher mean titer after the 2^nd^ boost. Four of the 6 macaques in the GM-CSF group developed titers >1000 after the 2nd boost whereas antibody titers in all 6 animals in the unadjuvanted group remained below 1000 (Figure 2B). These results show that PMED HA DNA vaccination was able to elicit significant levels of neutralizing HA-specific antibodies in the serum of immunized macaques. Furthermore, co-delivery of rhesus GM-CSF as a genetic adjuvant with the HA DNA vaccine accelerated the appearance of, and significantly increased the magnitude of, neutralizing antibodies in the sera of immunized animals.

### GM-CSF increases T cell responses in the blood

**Figure 3:**
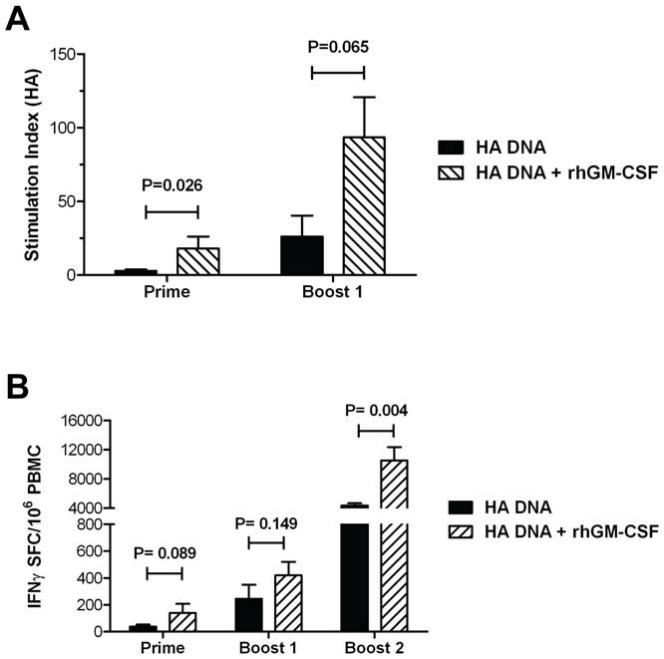
Co-delivery of the GM-CSF genetic adjuvant enhances HA-specific T cell responses in the peripheral blood of PMED HA DNA vaccinated animals. PBMCs were isolated from the blood of macaques at the indicated times following the initial vaccination with HA DNA (black bars) or HA DNA+rhGM-CSF (hatched bars). (**A**) T cell proliferative responses in vaccinated macaques were measured by incubating PBMCs with recombinant A/New Caledonia/20/99 HA protein (1 µg/well) for 6 days prior to the addition of 1 µCi tritiated thymidine. Bars represent the mean (± SEM) stimulation index calculated for each group of animals. (**B**) The number of HA-specific T cells present in the blood of macaques after HA DNA vaccine priming, the first boost, and second boost was measured by IFN-γ ELISPOT assay. Indicated P values were determined using the Mann-Whitney U test (two-tailed).

T cell responses play an important role in clearing influenza infections and in the development of a protective antibody response. To determine the impact of GM-CSF on T cell responses, lymphoproliferation (LPR) was measured in the blood after the first and second vaccine dose following stimulation with recombinant HA protein to determine the overall magnitude of the T cell response independent of a specific cytokine effector function. As shown in Figure 3A, only the GM-CSF adjuvanted group exhibited a measurable HA-specific T cell proliferative response following the prime. In addition, although both the GM-CSF-adjuvanted and unadjuvanted groups developed strong HA-specific T cell proliferative responses after the first boost, co-delivery of GM-CSF DNA notably enhanced the LPR in the blood.

As another means of measuring the T cell response elicited in the GM-CSF-adjuvanted and unadjuvanted vaccine groups, an ELISpot assay was used to enumerate the T cells in the blood of vaccinated animals secreting IFN-γ in response to stimulation with pools of overlapping peptides derived from the full length A/New Caledonia/20/99 HA protein. Similar to that observed with serum antibody responses, the number of IFN-γ secreting HA-specific T cells in blood was found to increase in both the unadjuvanted and GM-CSF adjuvanted groups following each dose of vaccine (Fig. 3B). However, the magnitude of T cell responses detected in the GM-CSF adjuvanted group was consistently higher than those detected in macaques vaccinated with the HA DNA vaccine alone. In particular, animals in the GM-CSF adjuvanted group exhibited over a 2-fold increase in the mean number of IFN-γ secreting HA-specific T cells in their peripheral blood following the 2^nd^ booster immunization as compared to animals in the unadjuvanted group (10,539 vs. 4,363, respectively). Taken together, the LPR and ELISpot results show that PMED HA DNA vaccination elicited strong HA-specific T cell responses in the blood of rhesus macaques that were significantly increased by the GM-CSF genetic adjuvant.

### PMED influenza DNA vaccines induce mucosal antibody responses in the respiratory tract

**Figure 4:**
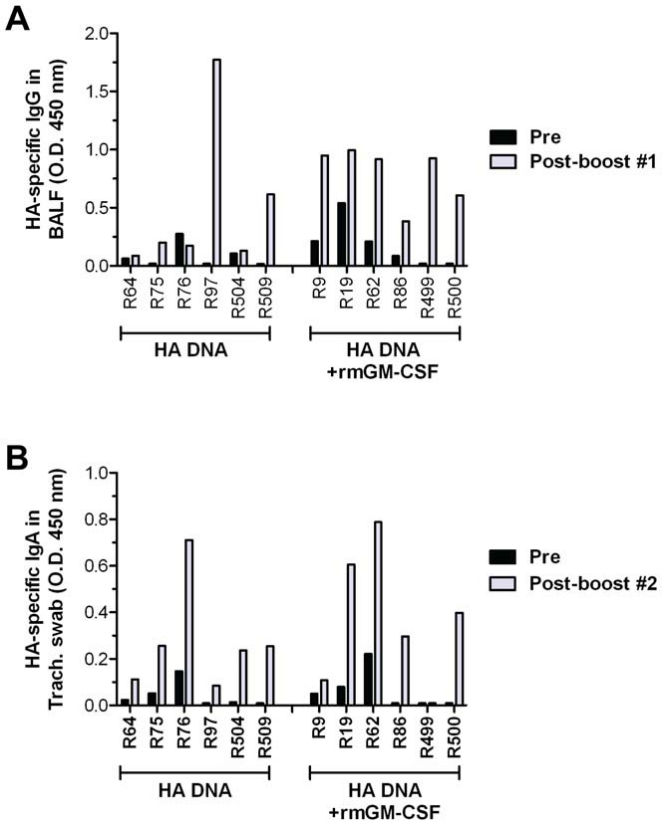
Induction of mucosal antibody responses in the respiratory tract of PMED HA DNA vaccinated macaques. Bronchoalveolar lavage fluid (BALF) and tracheal swab samples were collected from macaques prior to immunization and again at 4 weeks following each boosting dose of the HA DNA vaccine, ± rhGM-CSF. IgG antibody responses in BALF (**A**) and IgA antibody responses in tracheal swabs (**B**) against the A/New Caledonia/20/99 hemagglutinin protein were detected by ELISA. Data are reported as the O.D. measured at 450 nm for each sample, diluted 1∶20 in PBS, from individual immunized animals.

Mucosal IgA and IgG responses in the bronchioalveolar lavage fluid (BALF) and nasal and tracheal secretions were analyzed by ELISA using recombinant influenza A/New Caledonia/20/99 HA protein as the capture antigen. Following the first booster immunization, HA-specific IgG responses were detected in BALF (Figure 4A), but not in tracheal or nasal secretions (data not shown). In contrast, HA-specific IgA responses were detected in tracheal (Figure 4B) and nasal secretions (data not shown), but not in the BALF (data not shown), following the second booster immunization. The presence of a dominant HA-specific IgA response in the upper respiratory tract and a dominant HA-specific IgG response in the lower lung of vaccinated macaques is consistent with the pattern of mucosal antibody induced following influenza infections in mice [44], [45]. A comparison of mucosal antibody responses between the two vaccine groups showed that mucosal HA-specific IgG responses were detected in the BALF from just 2 out of the 6 macaques immunized with HA DNA alone (R97 and R509) whereas all 6 macaques in the GM-CSF group exhibited significant levels of mucosal IgG in their lungs. This outcome correlates with a more rapid production of HA-specific serum antibodies in the GM-CSF group (Figure 2) and suggests that the presence of HA-specific IgG in the BALF following PMED DNA immunization may result from the transudation of IgG from the serum into the lung mucosa [46], [47]. Mucosal HA-specific IgA responses were detected in tracheal secretions from all vaccinated macaques, with no significant difference noted between the HA DNA only and GM-CSF adjuvanted vaccine groups (Figure 4B). Analysis of the relative concentrations of total mucosal IgG (BALF) and IgA (tracheal secretions) by ELISA showed no differences in total mucosal immunoglobulin concentrations between mucosal samples collected before and post vaccination from each individual animal and no difference between the unadjuvanted and GM-CSF adjuvanted vaccine groups (data not shown) confirming that the observed increases in HA-specific mucosal antibody in the BALF and trachea were due to the effects of the vaccines. Together, these results demonstrate that PMED immunization was capable of inducing HA-specific mucosal antibody responses in both the upper and lower respiratory tract of nonhuman primates but this effect appeared not to be significantly impacted by co-administration of the GM-CSF genetic adjuvant.

### GM-CSF increases the magnitude and breadth of mucosal T cell responses in the lung and gut

**Figure 5:**
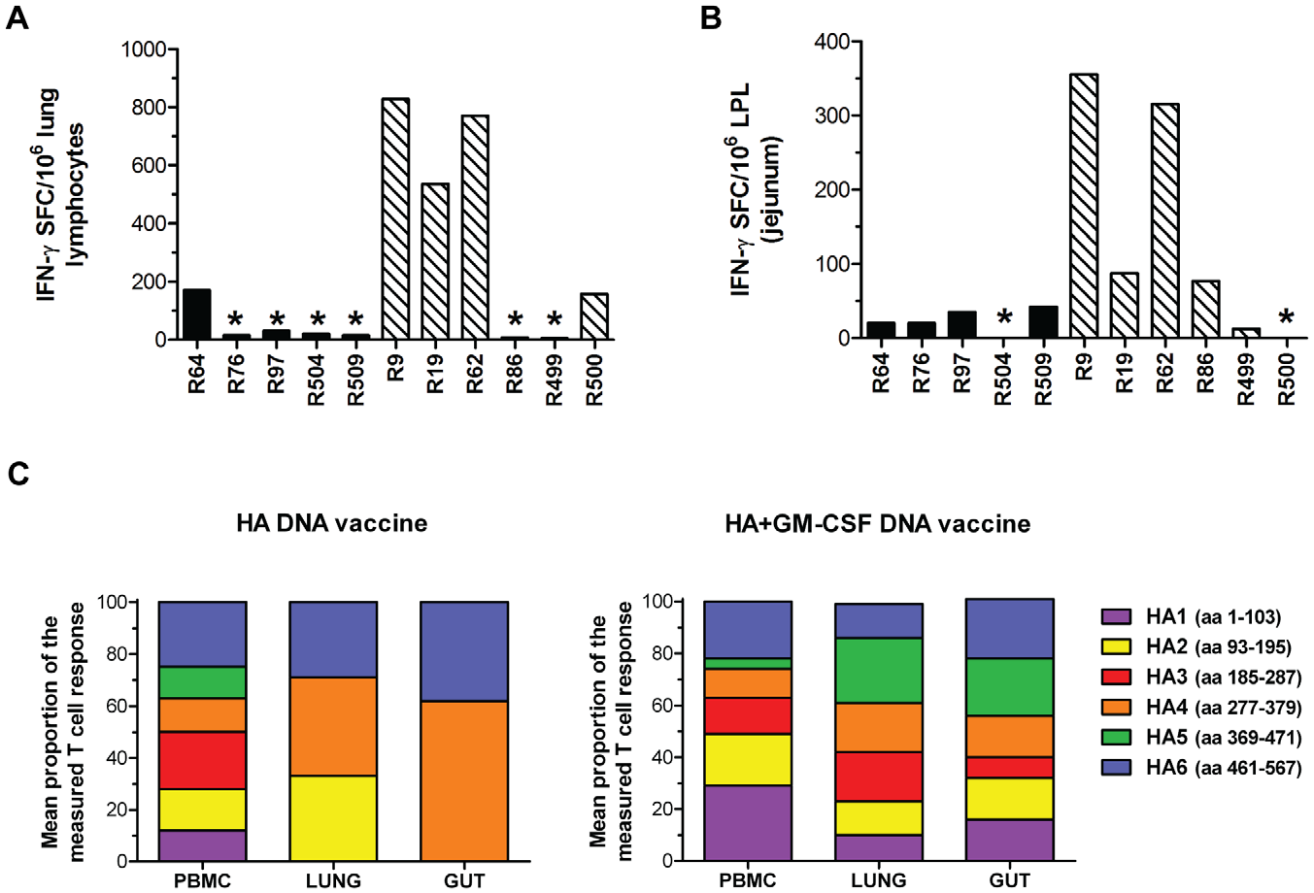
The GM-CSF genetic adjuvant increases both the magnitude and breadth of mucosal T cell responses elicited in the lungs and guts following PMED HA DNA vaccination. HA-specific T cell responses in the (**A**) lung and (**B**) gut mucosa of macaques were determined by IFN-γ ELISpot assay 4–11 weeks after the final vaccination with HA DNA (solid bars) or HA DNA+GM-CSF (hatched bars). Individual bars represent the peak number of HA-specific IFN-γ T cells detected in the jejunum and lung tissue of individual animals 4–11 weeks after the final vaccination. (**C**) Breadth of the IFN-γ T cell response in PBMC, lung, and gut. HA-specific T cell responses in the indicated tissues were measured using a standard IFN-γ ELISpot assay with 6 individual pools of overlapping peptides (11 amino acid overlaps, 103 15-mers per pool) comprising the entire amino acid sequence of the influenza A/New Caledonia/20/99 HA protein. The percent contribution of each peptide-pool specific response to the total response was determined by dividing the mean number of IFN-γ spot forming cells (SFC) measured against each individual peptide pool by the sum of the response against all peptide pools. Results represent the average of 2 time-points tested after the 3^rd^ DNA dose (weeks 19 and 23). *Measurement below positive threshold level for the assay.

Delivery of vaccines into the skin by PMED has been shown to induce T cell responses in both the blood and mucosal compartments [17], [48]. Accordingly, we next examined the capacity of PMED HA DNA vaccination to elicit HA-specific T cell responses in the lung and gut mucosa of immunized macaques. HA-specific T cell responses in the lungs and jejunum of vaccinated macaques 8–10 weeks following the final DNA immunization (weeks 24–26 post-prime) were measured using an IFN-γ ELISpot assay. As shown in Figure 5A, only 1 out of 5 macaques from the unadjuvanted DNA vaccine group developed a modest (<200 spot forming cells per10^6^ lung lymphocytes) HA-specific mucosal T cell response in the lung (Figure 5A, black bars). In contrast, HA-specific mucosal T cell responses were detected in the lung in 4 of 6 macaques in the GM-CSF adjuvanted group (Figure 5A, hatched bars) with 3 of these animals exhibiting strong mucosal T cell responses that exceeded 500 spot forming cells per 10^6^ lung lymphoctyes. In the gut, mucosal T cell responses were detected in all but 1 macaque in each DNA vaccine group but similar to the lung, gut T cell responses in several animals immunized with the GM-CSF adjuvant reached substantially higher levels than levels observed in unadjuvanted group (Figure 5B). Overall, the combined mucosal T cell responses that developed in the lungs and guts of animals immunized with the GM-CSF adjuvanted HA DNA vaccine were significantly higher than in the unadjuvanted HA DNA vaccine group (P = 0.031, Mann-Whitney U test), supporting the concept that GM-CSF can function as a mucosal adjuvant for DNA vaccines administered into the skin.

The breadth of the anti-viral T cell repertoire elicited following infection or vaccination is thought to be a critical component in protection against drifted strains of influenza virus [49]. To determine if co-administration of the GM-CSF genetic adjuvant influenced the breadth of the HA-specific T cell response elicited in vaccinated macaques, lymphocytes isolated from the blood, lungs, and guts of immunized animals were stimulated with 6 separate pools of overlapping HA peptides and analyzed for responses against each pool in an IFN-γ ELISpot assay. Each HA peptide pool consisted of 23 to 24 15-mer peptides, overlapping by 11 amino acids, spanning the entire sequence of the HA protein. As shown in Figure 5C, animals from both the unadjuvanted and GM-CSF adjuvanted vaccine groups exhibited equally broad T cell responses against each of the 6 peptide pools in the peripheral blood. In the lung and gut mucosa, macaques immunized with HA DNA alone exhibited mucosal T cell responses against just 2–3 of the 6 total peptide pools (left panel), indicating that the repertoire of HA-specific T cells induced in the mucosa of rhesus macaques immunized with the unadjuvanted vaccine was narrower than the repertoire of T cells detected in peripheral blood. Mucosal T cell responses in the gut and lung of the unadjuvanted group were dominated by responses against peptides in HA4 (aa 277–379) and HA6 (aa 461–567), suggesting a similarity in the repertoire of T cells elicited in these 2 distinct mucosal compartments. In contrast, macaques immunized with the GM-CSF-adjuvanted HA DNA vaccine (right panel) exhibited a broader repertoire of mucosal T cell responses against all 6 of the HA peptide pools that was more similar to the repertoire of T cells detected in the peripheral blood. Together, these results suggest that co-delivery of the GM-CSF genetic adjuvant was able to increase both the magnitude and breadth of the HA-specific T cell response induced in the mucosa following PMED HA DNA vaccination. These results also suggest that the use of GM-CSF DNA as an adjuvant may overcome barriers in compartmentalization of immune responses following vaccination between the periphery (blood) and mucosa.

### Impact of GM-CSF DNA co-delivery on the induction of polyfunctional of HA-specific T cells in the periphery and mucosa of PMED HA DNA vaccinated macaques

**Figure 6:**
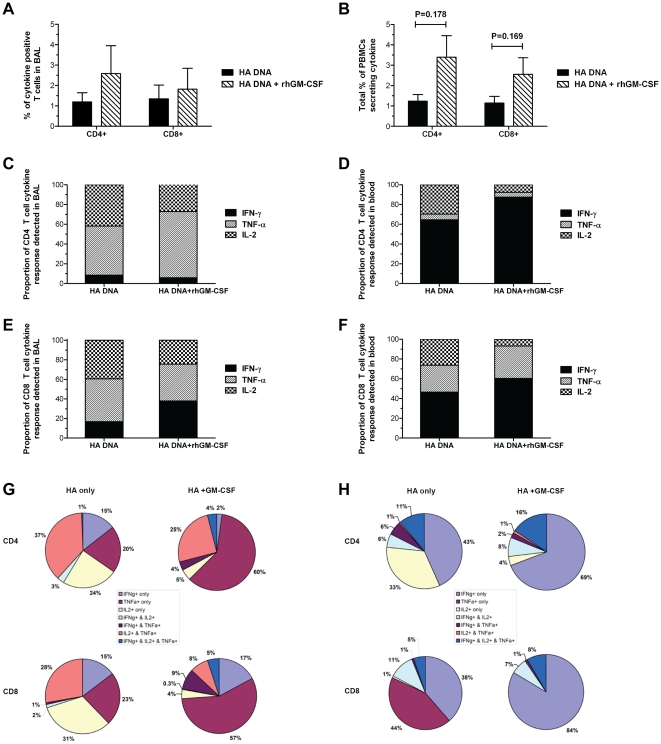
Generation of multifunctional T cells in the peripheral blood and lungs of macaques following PMED HA DNA vaccination. Intracellular cytokine staining was performed on BALF collected from both vaccine groups at 4 weeks after the first boost (panels on left), as well as PBMCs from both vaccine groups collected 4 weeks after the second boost (panels on right) to measure the expression of IFN-γ, TNF-α, and IL-2 by CD4+ and CD8+ T cells after stimulation with overlapping peptides derived from the A/New Caledonia/20/99 HA protein. Bar charts show the mean total percentage (+/− SEM) of CD4+ and CD8+ T cells in the lungs (**A**) and blood (**B**) of animals from the unadjuvanted (black bars) and GM-CSF adjuvanted (hatched bars) found to express IFN-γ, TNF-α, or IL-2 following HA peptide stimulation. Indicated P values were determined using the Mann-Whitney U test (two-tailed). Stacked bar charts show the mean proportion of cells producing IFN-γ (black), TNF-α (gray), or IL-2 (hatched) to the total HA-specific CD4+ and CD8+ T cell response detected in the lung (**C**, **E**) and blood (**D**, **F**) of animals from the unadjuvanted and GM-CSF adjuvanted vaccine groups. Pie charts show the proportion of HA-specific CD4+ and CD8+ T cells in the lung (**G**) and blood (**H**) from both vaccine groups positive for the different combinations of one, two, or three cytokines.

Recent studies indicate that polyfunctional T cells correlate with durable protective immunity [50]. Furthermore, the quality of the local virus-specific T cell responses in the lung can influence the clearance of respiratory infections, including influenza [51]. We therefore sought to characterize the impact of co-delivering GM-CSF DNA on nature and quality of the influenza HA-specific T cell functional response in the lung and blood of the vaccinated macaques. The relative contribution of HA-specific CD4+ and CD8+ T cell subsets to the lung T cell response after the first booster immunization and the blood T cell response after the second booster immunization was determined by intracellular cytokine staining of T cells stimulated with influenza A/New Caledonia/20/99 HA peptide pools. Animals immunized with the GM-CSF-adjuvanted DNA vaccine exhibited a trend toward a higher frequency of HA-specific CD4+ T cells producing either IFN-γ, TNF-α, or IL-2 cytokines in both the lung (Figure 6A) and blood (Figure 6B) when compared to animals immunized with the HA DNA vaccine alone. The GM-CSF adjuvanted group showed over a two-fold increase in the mean frequency of cytokine-producing HA-specific CD4+ T cells in both the lung (2.58% vs. 1.19% ) and blood (3.39% vs. 1.23%) when compared to the unadjuvanted group, although these difference fell short of statistical significance due to the small number of animals per group. Similarly, the frequency of cytokine-producing HA-specific CD8+ T cells in the lung and blood tended to be higher in the GM-CSF-adjuvanted vaccine group when compared to the unadjuvanted group but this increase was not statistically significant (Figures 6A and B).

We further characterized the nature of the T cell responses induced by PMED HA DNA vaccination by assessing the relative contribution of each cytokine (IFN-γ, TNF-α, or IL-2) to the total HA-specific CD4+ and CD8+ T cell responses. Figure 6C shows that for both vaccine groups, TNF-α and IL-2 dominated the CD4+ T cell cytokine response detected in the lung. This result was markedly different from the pattern of cytokine expression by HA-specific CD4+ T cells detected in the blood where the response for both groups was dominated by IFN-γ (Figure 6D). For both the lung and blood, co-delivery of GM-CSF DNA with the HA DNA vaccine augmented expression of the predominant cytokine (TNF-α in the lung, IFN-γ in the blood) in the CD4+ T cell response. In characterizing the nature of the CD8+ T cell response induced by vaccination, HA-specific CD8+ T cells producing IFN-γ, TNF-α, or IL-2 were detected in the lung (Figure 6E) and blood (Figure 6F) of both vaccine groups. In both the lung and blood, co-delivery of GM-CSF DNA tended to reduce the proportion of CD8+ T cells expressing IL-2 and HA-specific CD8+ T cells producing IFN-γ and TNFα comprised a greater portion of the total CD8+ T cell response in this group.

We next utilized Boolean analyses to determine the frequency of HA-specific CD4+ and CD8+ T cells expressing one, two, or three cytokines in the lung and blood of both vaccine groups. Figure 6G shows that immunization with the HA DNA vaccine alone induced a HA-specific CD4+ T cell response in the lung that was comprised almost entirely of T cells expressing a single cytokine (59%), with an almost equal distribution of cells expressing IFN-γ, TNF-α, or IL-2. The majority of the remaining HA-specific CD4+ T cells in the lungs of HA DNA vaccinated animals (40%) were found to express two cytokines, with the pre-dominant phenotype being co-expression of IL-2+ and TNF-α+. Co-administration of the GM-CSF genetic adjuvant induced a modest, but discernable increase, in the frequency of HA-specific CD4+ T cells in the lung expressing all three cytokines (4% vs. 1%). However, the main impact of GM-CSF on the nature of the CD4+ T cell response in the lung of HA DNA vaccinated animals was centered on the mono-functional cells. In contrast to the unadjuvanted group where monofunctional responses were distributed fairly equally between the cytokines, the majority of mono-functional HA-specific CD4+ T cell response in the lungs of animals immunized with GM-CSF expressed TNF-α (60%; Figure 6G).

Immunization with the HA DNA vaccine alone elicited mostly HA-specific CD8+ T cells in the lungs expressing one cytokine (69%; Figure 6G), with the remaining population (31%) found to express two cytokines. As with the HA-specific CD4+ T cell response, the majority of the dual-cytokine producing CD8+ T cells in the lungs of animals from the unadjuvanted group expressed IL-2 and TNF-α (Figure 6G). CD8+ T cells expressing all three cytokines were not observed in the lungs of macaques immunized with HA DNA vaccine alone. Similar to the effect on the CD4+ T cell response, co-delivery of GM-CSF DNA increased the frequency of mono-functional CD8+ T cells in the lung expressing TNF-α when compared to the unadjuvanted group (57% vs. 23%). This increase corresponded to a large decline in the frequency of mono-functional CD8+ T cells expressing IL-2 (4% vs. 31%). However, similar to what was observed with the CD4+ T cell response in the lung, co-administration of GM-CSF DNA resulted in the appearance of polyfunctional HA-specific CD8+ T cells expressing all 3 cytokines, IFN-γ, TNF-α, and IL-2 (5%; Figure 6G). GM-CSF also appeared to alter the phenotype of dual functional CD8+ T cells in the lung. In contrast to the predominance of HA-specific CD8+ T cells expressing IL-2/TNF-α in the lungs of animals from the unadjuvanted group, the population of dual functional CD8+ T cells in the lungs of animals from the GM-CSF adjuvanted group was comprised almost evenly of IL-2+/TNF-α+ and IFN-g+/TNF-α+ CD8+ T cells (Figure 6G).

The pattern of cytokine expression by HA-specific T cells in the blood (Figure 6H) was found to be markedly different from the pattern we observed in the lung mucosa. Immunization with the HA DNA vaccine alone induced a HA-specific CD4+ T cell response in the blood that was comprised almost entirely of mono-functional T cells expressing either IFN-γ or IL-2 (43% and 33%, respectively; Figure 6H). The remaining population of HA-specific CD4+ T cells in the lungs of HA DNA vaccinated animals was split almost evenly into dual- and triple-cytokine expressing cells (13% and 11%, respectively). As with the CD4+ T cell response in the lung, the main impact of GM-CSF on the nature of the CD4+ T cell response in the blood of HA DNA vaccinated animals was an increase in the proportion of mono-functional cells. The mean frequency CD4+ T cells in the blood of vaccinated animals expressing IFN-γ alone increased from 43% in the unadjuvanted group to 69% in the GM-CSF adjuvanted group (Figure 6H). Furthermore, the increase of IFN-γ+ mono-functional CD4+ T cells in the blood of the GM-CSF adjuvanted group corresponded to a large decline in the frequency of mono-functional CD8+ T cells expressing IL-2 when compared to the unadjuvanted group (4% vs. 33%).

Similar to the CD4+ T cell response, immunization with the HA DNA vaccine alone elicited a HA-specific CD8+ T cell response in the blood that was comprised almost entirely of mono-functional T cells expressing either IFN-γ or IL-2 (38% and 44%, respectively; Figure 6H). The remaining population of HA-specific CD8+ T cells in the lungs of HA DNA vaccinated animals was made up of dual- and triple-cytokine expressing cells (13% and 5%, respectively), with the majority of dual-functional CD8+ T cells expressing IFN-γ and IL-2. Co-administration of the GM-CSF DNA induced a significant increase in the frequency of mono-functional HA-specific CD8+ T expressing IFN-γ in the lungs of vaccinated macaques (mean of 84% in the GM-CSF adjuvanted group vs. mean of 38% in unadjuvanted group). The large increase in the frequency of IFN-γ+ mono-functional CD8+ T cells corresponded to a loss of detectable mono-functional CD8+ T cells expressing TNF-α in the blood of vaccinated macaques from the GM-CSF adjuvanted group. Co-delivery of GM-CSF DNA appeared to slightly decrease the overall frequency of dual-functional CD8+ T cells in the blood of vaccinated macaques but modestly increase in the frequency of triple cytokine expressing HA-specific CD4+ (16% in the GM-CSF adjuvanted group vs. 11% in the unadjuvanted group) and CD8+ T cells (8% in GM-CSF adjuvanted group vs. 5% in unadjuvanted group). Taken together, results from our Boolean analyses reveal considerable differences in the nature and quality of the T cell responses induced in the blood and lung mucosa following PMED HA DNA immunization. Furthermore, co-delivery of the GM-CSF DNA altered the functionality of HA-specific T cells but this effect markedly differed between the two compartments.

## Discussion

In this study we assessed the systemic and mucosa immunogenicity of an influenza (A/New Caledonia/20/99) HA DNA vaccine administered by particle-mediated epidermal delivery (PMED or gene gun) in rhesus macaques. In addition, we evaluated whether co-administration of the GM-CSF genetic adjuvant could augment the immunogenicity of the PMED HA DNA vaccine. The data show that PMED DNA vaccination induced significant levels of hemagglutination inhibiting (HI) antibodies and multi-functional HA-specific CD4+ and CD8+ T cell responses in the periphery of macaques after priming and that these responses increased after each subsequent boosting dose. In addition, PMED HA DNA vaccination induced detectable levels of HA-specific mucosal antibodies and T cells in the lung and gut-associated lymphoid tissues of vaccinated macaques. Importantly, our results reveal that co-delivery of plasmid DNA encoding the rhesus macaque GM-CSF gene could significantly enhance both the systemic and mucosal responses induced by PMED HA DNA vaccination.

PMED DNA vaccines possess a number of features which may allow them to overcome many of the perceived shortcomings of the more conventional vaccination technologies. In addition to antibody, PMED DNA vaccines induce T cell responses against epitopes that are conserved across variant virus strains and may thereby provide a second level of protection from illness if the antibody response fails [52] and improve protective efficacy in the elderly [39]. Further, the ability of PMED DNA vaccines to induce mucosal immune responses could provide additional benefit by limiting viral replication at the site of exposure. PMED DNA vaccines have consistently demonstrated greater efficiency in human clinical trials than DNA vaccines injected by needle or liquid jet propulsion into the muscle. In these studies, PMED elicited both T cell responses and protective levels of antibody in 100% of vaccinated subjects [28], [40]. However, in comparing results from hepatitis B and influenza PMED DNA vaccine clinical trials where an existing licensed vaccine is available, antibody responses induced by PMED arose more slowly, required more doses, and/or reached titers that were lower than those historically observed with licensed killed or recombinant protein vaccines [28], [29], [40].

The present study utilized the same disposable clinical prototype PMED device (ND10) previously tested for immunogenicity in a clinical trial [29]. In this clinical study, subjects were vaccinated by PMED with a trivalent (H1+H3+B) influenza HA DNA vaccine and then challenged with an H3 strain of influenza. Vaccination was safe and well-tolerated and elicited protection from challenge in approximately 50% of subjects, providing the first proof-of-principle of a DNA vaccine affording protection in humans. However, efficacy was lower than reported for licensed vaccines (70%) indicating that greater potency was required before the PMED technology could match conventional influenza vaccines. Our results here show that GM-CSF increased neutralizing antibody titers to levels that substantially exceeded levels known to afford protection against influenza in humans and significantly accelerated seroconversion such that antibody responses were detected in 100% of the macaques after only a single immunization. This outcome strongly suggests that GM-CSF may provide a means to overcome the problems of lower immunogenicity and slower development of antibody responses observed with unadjuvanted influenza DNA vaccines in the clinic when compared to existing licensed vaccine approaches.

GM-CSF was also found to both increase the induction of peripheral and mucosal CD4+ and CD8+ T cell responses following PMED DNA vaccination and influence the functionality of those responses. We also observed that PMED DNA immunization, with or without GM-CSF, elicited high frequencies of mono-functional CD4+ and CD8+ T cells. IFN-γ was the predominant cytokine expressed by T cells in the blood, whereas TNF-γ dominated responses in the lung. It is currently not known what role TNFα plays in controlling influenza infection but, like IFNγ, TNFα is known to have direct antiviral effects and is a dominant cytokine produced during influenza virus infections [51]. In addition, a recent study showed that induction of TNFα responses may be important in the development of more effective B and T cell recall responses against influenza suggesting that a vaccine that enhances T cells with TNFα effector function may influence its ability to confer durable immunity [53]. We also found that GM-CSF increased the frequency of triple cytokine-producing T cells in vaccinated macaques, an outcome that is consistent with a previous effect of GM-CSF on an HIV DNA vaccine [36]. However, our study shows that this effect was more pronounced in the lung than in the blood. This finding is critical, as multi-functional T cell responses have been correlated with improved vaccine-mediated protection in the blood [50], [54] and may similarly influence mucosal protection against a disseminated infection in the lung. An explanation for why the GM-CSF genetic adjuvant apparently had diverse effects on the functionality of T cell responses in the blood and lung of PMED HA DNA vaccinated animals is unclear, but we hypothesize that it is likely related to different effects of GM-CSF on antigen presentation. Encounters with mature, Ag-bearing dendritic cells within draining lymph nodes facilitate T cell activation, proliferation, and differentiation to effector cells before migration out to the periphery and mucosa. Moreover, recent studies have suggested that T cell functionality, particularly cytokine production, is dependent on the stimulating APC [55].

Previous findings by Lai et al. [33] showing IgA antibody responses in rectal secretions of macaques immunized with a GM-CSF-adjuvanted DNA vaccine provided the first indication that GM-CSF may be able to enhance the mucosal immunogenicity of DNA vaccines. However, the impact of GM-CSF on the induction of mucosal antibody in the lung and T cell responses in the lung or gut has not been previously studied. We and others have shown that unadjuvanted SIV and influenza PMED vaccines induced mucosal responses in mice, monkeys, and/or swine that were associated with enhanced protection against either intranasal challenges with influenza [14], [18], [48] or rectal challenges with SIV [17]. We were therefore particularly interested to determine if GM-CSF influenced the ability of PMED to induce mucosal responses. The present study is the first to show that GM-CSF can function as a strong mucosal adjuvant when co-administered to the skin with an influenza DNA vaccine enhancing both the frequency of mucosal antibody detected in the lung and the magnitude, breadth, and function of T cell responses in the lung and gut. Skin immunization has been shown to be superior to parenteral immunization for induction of both systemic and mucosal immune responses [56], [57], but the mechanisms whereby immune responses are induced in mucosal compartments distant from the initial skin site of vaccine administration are still unclear. Studies in mice suggest an important role for antigen presenting cells in the skin in the induction of mucosal responses [20], [22], [58], and GM-CSF is a key cytokine influencing the maturation and migration of APC including epidermal Langerhans cells [59]. Thus, two possibilities currently under study in our laboratory is that GM-CSF enhances mucosal immunogenicity of skin-delivered PMED influenza DNA vaccines by increasing the migration of antigen presenting cells bearing skin-derived antigen to mucosal compartments [20] and/or enhances the ability of skin-derived APC to imprint a mucosal homing phenotype on stimulated T cells [60].

Interestingly, our results showed that GM-CSF increased the breadth of peptide-specific T cell responses in the gut and lung mucosa but not the blood, an outcome that supports both possible mechanisms. A broader T cell response in the mucosa may enhance control of viral replication by targeting multiple epitopes expressed by the homologous virus, as well as potentially shared HA epitopes among drifted or shifted influenza viruses. Although we did not perform fine epitope mapping of the HA-specific T cell responses detected in vaccinated animals, it is noteworthy that the level of sequence homology between the HA protein of A/New Caledonia/20/99 and the HA proteins of drifted strains of H1N1 virus may be high enough to support the existence of cross-reactive T cell epitopes. For example, sequence alignment shows that the HA protein of A/New Caledonia/20/99 shares 97% sequence homology with A/Brisbane/59/2007, 97% homology with A/Solomon Islands/03/2006, and 80% homology with A/California/04/2009 [61]. In considering the potential capacity of PMED HA DNA vaccination to elicit cross-reactive T cell responses able to recognize HA proteins from shifted strains of virus, it is notable that the addition of the GM-CSF adjuvant increased T cell responses against regions of the A/New Caledonia/20/99 HA protein found to exhibit sequence homology with the HA protein of the H5N1 A/Vietnam/1203/04 avian strain of influenza. ClustalW2 alignment analyses revealed ∼80% sequence homology between these two strains for peptide pool HA5 and ∼75% sequence homology for peptide pool HA6 [61]. Challenge studies in a relevant mucosal infection model will be needed to determine the significance of this effect with respect to improving vaccine protection against both drifted and shifted strains of influenza. It will also be important to examine the effect of GM-CSF on the T cell responses against more conserved influenza viral antigens (i.e., nucleoprotein and M2e) that could further improve cross-protection against a wide variety of influenza A viruses.

Previous clinical trials using injections of GM-CSF protein as an adjuvant have been largely disappointing with some trials showing no enhancement of systemic T cell or serological immunity to co-delivered vaccines [62], [63] although these trials did not assess mucosal responses. The beneficial systemic and mucosal effects of GM-CSF in rhesus macaques in the present study may be due to co-delivery of GM-CSF as a plasmid with the DNA vaccine directly into antigen presenting cells in the skin [6]. In this setting, the GM-CSF adjuvant is expressed at the same site, in the same cells, and with similar kinetics as the target vaccine antigen and may have more direct effects on enhancing antigen presentation and T cell activation. Whether these effects translate from rhesus to man can only be assessed by clinical trials.

Mucosal immune responses can contribute to vaccine efficacy by blocking or limiting viral replication at the site of exposure and the discovery of new vaccines that elicit mucosal immunity is currently of significant interest [64], [65]. To date, most experiments in nonhuman primates have focused on measuring vaccine responses and immune correlates of protection primarily in the blood. Recent studies have started to investigate the capacity, especially for candidate AIDS vaccines (HIV or SIV), to induce mucosal responses in the lung [66] or gut [64] as a means to determine the possible contribution or correlation of these responses to protection. The study reported here provides a broad comparison of the magnitude, nature, and breadth of T cell responses induced by a DNA vaccine in the blood, lung, and gut of rhesus macaques and shows there can be significant differences in each compartment. These findings emphasize that immune responses measured only in the blood may not fully represent a vaccine's immunogenicity and potential for protective efficacy against mucosally transmitted diseases. A vaccine technology capable of inducing both antibody and improved anti-influenza T cell responses, especially at mucosal surfaces where influenza infection takes place, may provide superior protection. A further potential advantage of this technology for pandemic preparedness is the speed with which a new DNA vaccine can be synthesised and manufactured. The time taken to deploy a novel influenza vaccine may have a critical influence on the mortality of a developing pandemic, and the avoidance of use of eggs, live viruses and mammalian cell culture in the manufacture of DNA vaccines may provide greater surety of production and a more rapid response than more conventional influenza vaccine technologies. The ease of manufacture together with the improved systemic and mucosal immunogenicity reported here support further development of particle-mediated DNA vaccines in combination with GM-CSF as a clinically-viable approach for improving vaccination against influenza.

## Materials and Methods

### Ethics Statement

The University of Pittsburgh takes responsibility for humane care and use of laboratory animals in all research projects including those awarded by the Public Health Service. They are committed to comply with the Principles for Use of Animals, the National Institute of Health Guide for the Care and Use of Laboratory Animals, the Provisions of the Animal Welfare Act, and other applicable laws and regulations which are consistent with the recommendations of the Weatherall report “The use of non-human primates in research”. The university's Statement of Assurance is on file with the PHS, Office for Protection from Research Risks (A3187-01). The University of Pittsburgh is accredited by the American Association for the Accreditation of Laboratory Animal Care International (AAALAC). All experimental manipulations were approved by the University's Institutional Animal Care and Use Committee (approval # 0711526). Animals were cared for by well established, competent clinical veterinary and animal caretaker staff. Animal discomfort and pain was alleviated by appropriate and routine use of anesthetic and/or analgesic agents.

### Influenza A/New Calendonia/20/99 HA DNA vaccine (pPML7800)

The HA coding sequence was synthesized at GeneArt (Regensburg, Germany) from the full-length amino acid sequence of the influenza A/New Caledonia/20/99 (H1N1) hemagglutinin (HA) protein obtained from the Influenza Sequence Database (http://www.flu.lanl.gov), using a codon usage pattern commonly found in human genes (http://www.kazusa.or.jp/eng/index.html). Plasmid pPML7800 was generated by inserting the codon-optimized H1 HA gene into an expression cassette (pPJV7563) that uses the human cytomegalovirus immediate early promoter. Additional sequences were included to improve expression, specifically the HBV pre-S2 5′ UTR, CMV exon ½ (consisting of the first two CMV IE exons spliced together by deletion of the natural intron), rat insulin intron A, HBV env enhancer, and rabbit beta globin poly A (rGpA).

Expression of the HA antigen from pPML7800 was confirmed in B16 melanoma cells (ATCC, Manassas, VA). Cells were transfected with pPML7800 using Lipofectin® (Invitrogen, Carlsbad, CA) according to manufacturers' instructions. At eighteen hours post transfection, cells were fixed with a 50/50 mixture of methanol/acetone and blocked with 5% dry milk/TBS (Bio Rad Laboratories, Melville, NY). The cells were probed with a 1/3,000 dilution of sheep antisera raised against New Caledonia (CBER, Rockville, MD) in 1% dry milk/TBS/0.05% Tween-20 (Sigma-Aldrich, St. Louis, MO), followed with a 1∶1,500 dilution of rabbit anti-sheep/biotin (KPL, Gaithersburg, MD) in 1% dry milk/TBS/0.05% Tween-20, and finally with a 1/1,800 dilution of streptavidin/HRP conjugate (KPL, Gaithersburg, MD) in TBS/0.1% Tween-20. Color was developed using tetramethylbenzidine substrate (TMB; Sigma). The reactions were stopped with 1N H2SO4, and 200 microliters were aliquoted to an ELISA plate and read at 450nm. Successful expression of the HA antigen in transfected B16 cells was confirmed by an absorbance reading six times greater than the reading obtained with non-transfected cells.

### Rhesus GM-CSF genetic adjuvant vector

The rhGM-CSF coding sequence for plasmid pPML7842 was synthesized at GeneArt (Regensburg, Germany) using the published amino acid sequence for *Macaca mulatta* GM-CSF [67]. The codon-optimized gene was inserted into expression cassette pPJV7563 as described for pPML7800. Expression of rhesus macaque GM-CSF from pPML7842 was confirmed in B16 melanoma cells following Lipofectin® transfection using a similar method to the one described above for pPML7800 substituting the use of a biotinylated anti-human GM-CSF monoclonal antibody (BioLegend, San Diego, CA) cross-reactive to rhesus macaque GM-CSF.

### Rhesus macaques

Adult male Indian origin rhesus macaques (*Macaca mulatta*) were obtained from an approved vendor. All procedures were performed under sedation with ketamine (Parke-Davis, Ann Arbor, MI) (10mg/kg). Bronchial alveolar lavages (BAL) were performed on anesthesized animals pre-oxygenated to induce blood oxygen saturation levels of >95%. Glycopyrolate at 0.01 mg/kg was administered intra-muscularly (IM) prior to the insertion of a pediatric bronchoscope. Immediately following insertion of the bronchoscope, 2–3 ml of 1% sterile Lidocaine was infused into the lung. To harvest the BAL fluid, 10 ml aliquots of sterile 0.9% bacteriostatic saline was infused into the lung via the bronchoscope and aspirated to remove. Oxygen was again administered immediately following the procedure to maintain blood oxygen saturation levels of >95%. Tracheal swabs were collected using a cotton tip applicator and the secretions collected were suspended in 0.5ml saline. Nasopharyngeal specimens were collected by instillation of 0.5 ml saline into each nare. Jejunal resections (10–15 cm) and lungs were collected at necropsy.

### ND10 PMED DNA vaccine immunizations

Plasmid DNA was precipitated onto 1–3 µm gold particles as previously described [40] at a rate of 1.8 µg pPML7800 and 0.2 µg pPML7842 (10∶1 ratio of DNA vaccine to adjuvant) per 1.0 mg of gold. Animals were sedated with ketamine (10 mg/kg; Parke-Davis, Ann Arbor, MI), the inner leg fur was clipped, and DNA-coated gold particles were accelerated into the skin of both the abdominal and inguinal lymph node regions using a single use, disposable ND10 clinical particle-mediated epidermal delivery (PMED) device (PowderMed, Ltd., Oxford, U.K.) to deliver the DNA directly into the cells of the epidermis as described [29]. DNA-coated gold particles were delivered at a helium pressure of 45 bar psi. Each actuation resulted in the delivery of 1.0 mg of gold and 2.0 µg of DNA into the epidermis. A single dose consisted of a 12.0 µg of total DNA (10.8 µg HA DNA vaccine and 1.2 µg GM-CSF genetic adjuvant) per immunization and was achieved by using 6 disposable devices to administer 6 actuations into the skin of the inner thigh. Each of the 3 doses was administered 8 weeks apart.

### Isolation of mononuclear cells from blood, lung, and jejunum

Rhesus macaque peripheral blood mononcuclear cells (PBMC) were isolated by Ficoll density gradient centrifugation, erythrocytes removed using ACK Lysing buffer (BioWhittaker, Walkersville, MD), and remaining cells washed 3 times with RPMI-1640 media (BioWhittaker) supplemented with 10% fetal bovine serum (Life Technologies, Gibco BRL) (R10). Bronchoalveolar lavage fluid (BALF) cell suspensions collected at necropsy were obtained by instilling and aspirating 150 ml of PBS into the main bronchus of each lung. BALF cells collected by either bronchoscope or at necropsy were pooled and concentrated by centrifugation, washed twice in HBSS (Ca^2+^/Mg^2+^-free; Cellgro/Mediatech; Fisher Scientific, Federal Way, WA), and then resuspended in R10 media. Mononuclear cells were isolated from jejunal resections using a mechanical and enzymatic (dispase) extraction protocol as described [17], [68], with mononuclear cells released from the lamina propria (lamina propia lymphocytes or LPL) then purified using Percoll gradient separation.

### Histology

Bead penetration into the skin of rhesus macaques was tested prior to vaccination in 2 macaques. One milligram of placebo gold (no DNA) was delivered into the skin. Each vaccine site was then removed by 8 mm punch biopsy 10 minutes following vaccination for cross sectioning. Skin biopsies were frozen in liquid nitrogen after embedding in PolyFreeze tissue freezing media (Polysciences, Inc., Warrington, PA). Frozen skin samples were cryostatically cut into 10 µm sections, fixed with a mixed solution of formalin/acetic acid/methanol, and then stained with Gill's Haematoxylin and Eosin (H&E) to allow visualization of gold particle penetration and distribution.

### Hemagglutination inhibition assay

RDE (Accurate Chemical & Scientific Corp., Westbury, NY) treated sera were analyzed for the presence of influenza A/New Caledonia/20/99 specific antibody using a hemagglutination inhibition (HI) assay as described [29]. Briefly, two-fold serial dilutions of RDE-treated sera were incubated with four hemagglutination units of influenza A/New Caledonia/20/99 virus propagated in 11-day old embryonated eggs followed by the addition of turkey erythrocytes. Serum HAI titers are reported from the average of duplicate tests as the reciprocal dilution of serum found to inhibit hemagglutination. A titer of 5 was assigned to those serum samples that failed to inhibit hemagglutination at the starting dilution of 1/10.

### ELISA

HA-specific IgG and IgA antibody levels in macaque nasal washes, tracheal swabs, and bronchoalveolar lavage fluid (BALF) were assessed by enzyme-linked immunosorbent assay (ELISA). RIA/EIA plates (Corning-Costar) were coated with 31 ng/well of recombinant baculovirus-expressed A/New Caledonia/20/99 (Protein Sciences, Meriden, CT) overnight at 4°C, blocked with 10% FBS in PBS for 1 h at room temperature, and then washed three times with wash buffer (PBS-T; phosphate-buffered saline containing 0.05% Tween 20). Two-fold serial dilutions of samples were added to the wells and plates were incubated for 2–3 hrs at room temperature. Following three washes with PBS-T, plates were incubated with horseradish-peroxidase conjugated goat anti-macaque IgG (1/5,000 dilution) or IgA (1/4,000 dilution) secondary antibodies (Nordic Immunological Laboratories, The Netherlands) for 1 h at room temperature. After three washes with PBS-T, TMB substrate (Sigma-Aldrich) was added to the wells for 30 min at room temperature. Color development was stopped by the addition of 1 N H_2_SO_4_, and the plates were read at 450 nm. ELISA data from tracheal swab and BALF samples are reported as the OD_450_ nm reading obtained from a 1/20 sample dilution in sterile PBS. The 1/20 sample dilution was found to yield the least amount of background in addition to absorbance values greater than the threshold level of our ELISA assay.

The relative concentrations of IgG and IgA immunoglobulin in mucosal samples were assessed by direct ELISA. RIA/EIA plates (Corning-Costar) were coated overnight with two-fold serial dilutions of BALF (starting at 1/5 in PBS) and tracheal swab (starting at 1/10 in PBS) samples collected from individual macaques before and after vaccination. Wells were blocked, washed, and then incubated with horseradish-peroxidase conjugated goat anti-macaque IgG or IgA secondary antibodies (Nordic Immunological Laboratories) as described above. TMB substrate was added after incubation with detecting antibody and three washes with PBS-T. Color development was stopped using 1 N H_2_SO_4_. Data are expressed as the OD_450_ nm reading obtained at the indicated serial dilution of sample.

### Lymphoproliferation assay

Lymphoproliferative responses were measured using ^3^H-thymidine assay as previously described [69]. Briefly, whole PBMCs were cultured in triplicate wells at 2×10^5^ cells per well in a total volume of 200 µl of AIM-V medium (Invitrogen), in the presence of 0.2 µg of recombinant A/New Caledonia/20/99 HA protein (Protein Sciences, Meriden) for 6 days at 37°C, 5% CO_2_. ^3^H-labeled thymidine (1.0 µCi, Perkin-Elmer, Waltham, MA) was then added to each well and harvested after 18–20 hrs onto 96-well Unifilter GC plates (Packard Bioscience, Meriden, CT). ^3^H-labeled thymidine incorporation was measured using a Topcount liquid scintillation counter (Packard Bioscience) and results expressed as stimulation indices (S.I.), defined as the ratio of the mean counts per minute (cpm) of wells incubated with HA antigen to the mean cpm of wells incubated with medium alone. Controls included media alone and concanavalin A (ConA, Sigma) used at a final concentration of 5 µg/ml.

### IFNγ Enzyme-Linked Immunospot (ELISpot) Assay

Cells isolated from peripheral blood, BALF, or jejunum were stimulated with 6 individual pools of peptides (1µg total peptide/ml) spanning the hemagglutinin (HA) protein of the A/New Caledonia/20/99 (H1N1) strain of influenza virus (Mimotopes, Victoria, Australia). Peptides were 15mers overlapping by 11 amino acids and each pool contained 23–24 individual peptides. Concanavalin A (Sigma) was used for positive control wells (5µg/ml final) and media supplemented with 80% DMSO for negative control wells. HA-specific T cells secreting IFN-γ were detected by ELISpot assay using paired anti-macaque IFN-γ monoclonal antibodies (U-Cytech-BV, The Netherlands) as described [40], [69]. Spot forming cells (SFC) were enumerated using an ImmunoSpot® Analyzer with CTL ImmunoSpot® Professional Software (Cellular Technology Ltd., Shaker Heights, OH). Results are expressed as the mean number of SFC in replicate wells containing peptide minus the mean number of SFC in wells containing media/80% DMSO. Responses significantly higher than background levels (twice the number of SFCs enumerated in wells containing untreated cells plus 10 spots) were considered positive.

### Intracellular Cytokine Staining

Cells isolated from peripheral blood (fresh or cryo-preserved PBMCs) or BALF (fresh) collected from immunized macaques were incubated with pools of over-lapping15-mer peptides (1 µg/ml of each peptide final) derived from the HA protein of the influenza A/New Caledonia/20/99 virus at a concentration of 10^6^ cells/well, together with 0.5 µg each of the anti-human CD28 (clone CD28.2) and anti-human CD49d (clone 9F10) co-stimulatory antibodies (both from BD Biosciences, San Jose, CA) in a total volume of 200 µl of AIM V media. Cells were incubated with 5 µg/ml of SEB (Sigma-Aldrich) as a positive control or media supplemented with 80% DMSO as a negative control. After incubating for 1 hr at 37°C, cells were treated with 10 µg/ml of Brefeldin A (Sigma-Aldrich) and incubated for 5 hrs at 37°C. Plates were then wrapped in foil, stored at 4°C overnight. Cells were then washed twice with FACS buffer (PBS containing 2% FBS) and stained with APC-Cy7 conjugated anti-rhesus CD8 (clone RPA-T8), PerCP conjugated anti-rhesus CD4 (clone L200), and Pacific Blue™ conjugated anti-rhesus CD3 antibodies (clone SP34-2) in AIM-V medium. Cells were then fixed using BD Cytofix/Cytoperm™ solution and permeabilized using BD Perm/Wash buffer™ (both from BD Biosciences). Permeabilized cells were then stained with PE conjugated anti-human IL-2 (clone MQ1-17H12), APC conjugated anti-rhesus TNF-α (clone MAb11), and PE-Cy7 conjugated anti-human IFN-γ (clone 4S.B3) antibodies in Perm/Wash buffer. Following intracellular staining, samples were washed with FACS buffer (PBS +0.5%BSA+0.05% sodium azide) and then resuspended in 4% paraformaldeyde (Electron Microscopy Solutions). Approximately 250,000 events were collected on a LSR II flow cytometer (BD Biosciences) and data was analyzed using FlowJo v7.5 software (Tree Star, Inc., Ashland, OR). Unless otherwise specified, all antibodies were obtained from BD Biosciences. All flow cytometric staining data are reported after subtraction of the background level of staining.

### Statistical analysis

Statistical analyses were performed using GraphPad 5.03 statistical software program. Where indicated, statistical significance was assessed using the two-tailed Mann-Whitney U test. P values ≤0.05 were considered significant.
